# NESP55, a novel chromogranin-like peptide, is expressed in endocrine tumours of the pancreas and adrenal medulla but not in ileal carcinoids

**DOI:** 10.1038/sj.bjc.6600924

**Published:** 2003-05-27

**Authors:** A-M Jakobsen, H Ahlman, L Kölby, J Abrahamsson, R Fischer-Colbrie, O Nilsson

**Affiliations:** 1 Lundberg Laboratory for Cancer Research, Department of Pathology, Sahlgrenska University Hospital, SE-413 45 Göteborg, Sweden; 2 Lundberg Laboratory for Cancer Research, Department of Surgery, Sahlgrenska University Hospital SE-413 45 Göteborg, Sweden; 3 Department of Pediatrics, Queen Silvia Children's Hospital, Sahlgrenska University Hospital, SE-413 45 Göteborg, Sweden; 4 Department of Pharmacology, University of Innsbruck, Austria

**Keywords:** NESP55, chromogranin, endocrine tumours, gastrointestinal tract, pancreas, adrenal gland

## Abstract

Neuroendocrine secretory protein 55, NESP55, is an acidic protein belonging to the chromogranin family. The distribution of NESP55 in human tumours is not known. The aim of the present study was to study the expression of NESP55 in human gastrointestinal, pancreatic and adrenal tumours. A total of 118 human endocrine and nonendocrine tumours were examined by immunocytochemistry, and compared to the expression of chromogranin A (CgA) in the same tumours. Pancreatic endocrine tumours (14 out of 25), pheochromocytomas (19 out of 19), and neuroblastomas (seven out of 14) expressed NESP55, with the same strong labelling pattern in both benign and malignant tumours. Expression of NESP55 in pancreatic endocrine tumours and pheochromocytomas was confirmed by Western and Northern blot analysis. Immunocytochemical analysis demonstrated no labelling in ileal carcinoids (zero out of 15), and adrenocortical adenomas (zero out of 15). The majority of gastrointestinal and pancreatic carcinomas were negative for NESP55, with focal staining observed in two out of 30 tumours. In contrast, CgA was present in all neuroendocrine tumours examined (25 out of 25 pancreatic endocrine tumours, 19 out of 19 pheochromocytomas, 14 out of 14 neuroblastomas and 15 out of 15 ileal carcinoids). Thus, the expression of NESP55 in endocrine tumours of the gastrointestinal tract, pancreas and adrenals differs from that of CgA. Neuroendocrine secretory protein 55 is found in a subset of neuroendocrine tumours showing differentiation towards adrenal chromaffin cells and pancreatic islets cells.

Neuroendocrine tumours may arise in several organs, and have a common set of genes and markers, for example, hormones, vesicle proteins and enzymes. These proteins can be used as markers to distinguish the neuroendocrine tumours from nonendocrine tumours. The identification of a neuroendocrine tumour phenotype carries important prognostic and therapeutic implications. Endocrine tumours of the gut and pancreas have much better prognosis and responsiveness to therapy than their nonendocrine counterparts. However, endocrine differentiation may indicate a very aggressive clinical behaviour and poor prognosis in a subset of tumours, such as the poorly differentiated endocrine carcinomas (PDEC). Consequently, there is a need for specific and sensitive markers of neuroendocrine differentiation in diagnostic histopathology.

Accurate demonstration of neuroendocrine differentiation in tumours requires a broad spectrum of diagnostic tools. Immunocytochemical demonstration of secretory granule/vesicle proteins such as chromogranin A (CgA) and synaptophysin is the method of choice (Lloyd and Wilson, 1983) in demonstrating neuroendocrine differentiation. The huge biological diversity of neuroendocrine tumours necessitates the use of multiple neuroendocrine markers in order to detect and characterise all types of neuroendocrine tumours. Chromogranins constitute a group of acidic proteins which are widely expressed in neuroendocrine tissues (Simon and Aunis, 1989; Winkler and Fischer-Colbrie, 1992). They are localised to large dense core vesicles. Members of the chromogranin family are CgA and CgB, secretogranin II and III, VGF and neuroendocrine secretory protein 55 (NESP55) (Laslop *et al*, 2000). The physiological functions of chromogranins are still poorly understood. However, CgA has been shown to regulate secretory granule formation (Kim *et al*, 2001), and chromogranins are also known to be cleaved by endopeptidases to smaller biologically active peptide fragments such as pancreastatin, vasostatin I and II, and secretoneurin (Laslop *et al*, 2000).

Neuroendocrine secretory protein 55, the latest discovered member of the chromogranin family, is an acidic protein with an *M*_r_ 55 000. It has been cloned from cultured bovine chromaffin cells (Ischia *et al*, 1997), rat pituitary (Weiss *et al*, 2000) and a mouse pancreatic islet cell line (Hayward *et al*, 1998b) as well as from a human pheochromocytoma (Weiss *et al*, 2000). The NESP55-gene is genomically imprinted (Hayward *et al*, 1998a) and transcribed exclusively from the maternal allele (Li *et al*, 2000). Northern blot experiments have revealed a complex pattern of NESP55 mRNA transcripts because of splicing events (Weiss *et al*, 2000). Neuroendocrine secretory protein 55 is highly conserved among mammalian species. It is comprised of 245 amino acids with a predicted molecular mass of 28 kDa (Ischia *et al*, 1997), and is post-translationally modified by the addition of keratan sulphate glycosamine glycan chains (Weiss *et al*, 2000). The primary amino-acid structure of NESP55 contains five pairs of dibasic amino-acid residues, at which NESP55 can be cleaved by endopeptidases. Proteolytic processing of NESP55 is tissue dependent and varies greatly (Weiss *et al*, 2000). In the bovine adrenal medulla, NESP55 is cleaved efficiently to intermediate-sized and smaller molecules such as the C-terminal octapeptide (GAIPIRRH) (Ischia *et al*, 1997). In contrast, very little processing has been observed in the rat central nervous system (Weiss *et al*, 2000).

In bovine tissues, NESP55 was found at highest concentrations in the adrenal medulla; lower concentrations were found in the anterior pituitary, posterior pituitary and brain, pancreas, serum and urine. Neuroendocrine secretory protein 55 was not detected by RIA in the thyroid gland, lung, liver, spleen and testis. Very low concentrations of NESP55 were found in the intestines (Lovisetti-Scamihorn *et al*, 1999). Thus, the distribution of NESP55 resembles that of chromogranin A, but it may be less widely distributed. Neuroendocrine secretory protein 55 is expressed in the brain in phylogenetically old areas such as the brain stem and the hypothalamus (Bauer *et al*, 1999), which are involved in the regulation of basic autonomic and endocrine function. The aim of the present study was to analyse the expression of NESP55 protein in neuroendocrine tumours of the gastrointerstinal tract, pancreas and adrenals, and to evaluate the relation between NESP55 expression, tumour type and biological behaviour.

## MATERIALS AND METHODS

### Tumour material

A total of 118 human tumours from the files of the Department of Pathology, Sahlgrenska University Hospital, were analysed by immunohistochemistry. The following tumours were investigated: ileal endocrine tumours (ileal carcinoids) (*n*=15), pancreatic endocrine tumours (*n*=25), neuroblastomas (*n*=14), pheochromocytomas (*n*=19), adrenocortical adenomas (*n*=15) as well as nonendocrine tumours (adenocarcinomas) of the gastrointestinal tract and pancreas (*n*=30). Fresh tumour material was obtained from 35 patients undergoing surgery and was subjected to Western blot analysis: ileal carcinoids (*n*=9), pancreatic endocrine tumours (*n*=9), pheochromocytomas (*n*=9) and nonendocrine gastrointestinal tumours (adenocarcinomas) (*n*=8). Northern blot was performed on pheochromocytomas and ileal carcinoids.

### Immunocytochemistry

Tumour tissues were fixed in buffered formalin, dehydrated and embedded in paraffin wax. Sections (3–4 *μ*m) were placed on positively charged glass slides, deparaffinised and rehydrated. After microwave treatment with EDTA–NaOH, pH 8, twice for 5 min (NESP55 and tyrosine hydroxylase (TH)) or citrate buffer, pH 6 (CgA and insulin), sections were rinsed, blocked and incubated with primary antibodies overnight. Details of primary antibodies are given in Table 1

**Table 1:** Antibodies used for immunocytochemistry

**Antibody**	**Species**	**Dilution**	**Clone**	**Cat. no.**	**Source**	**Ref.**
Anti-NESP55	Rabbit	1 : 100	—	—	Reiner Fischer-Colbrie, Innsbruck, Austria	Ischia *et al* (1997)
Anti-CgA	Mouse	1 : 1000	LK2H10	1199021	Boehringer Mannheim, Mannheim, Germany	Lloyd and Wilson (1983)
Anti-TH	Mouse	1 : 150	2/40/15	1017381	Boehringer Mannheim, Mannheim, Germany	Rohrer *et al* (1998)
Anti-insulin	Guinea pig	1 : 100	—	A0564	DAKO A/S, Copenhagen	—

NESP55=neuroendocrine secretory protein, CgA=chromogranin A, TH=tyrosine hydroxylase.

. Neuroendocrine secretory protein 55 antisera were raised in rabbits immunised with a synthetic C-terminal fragment of bovine NESP55 (GAIPIRRH) coupled to keyhole limpet haemocyanin. The antibody reacts with bovine tissue which contains the NESP55 protein (Ischia *et al*, 1997). Bound antibodies were visualised using an indirect immunoperoxidase technique (EnVision+™, cat. no. K4002/rabbit, K4000/mouse; DAKO, Denmark). Diaminobenzidine (DAB) was used as chromogen. After counterstaining with Mayer's haematoxylin, sections were dehydrated and mounted. Slides were coded and evaluated by three observers. Immunolabelling was graded as follows: 0=<1% positive tumour cells, 1+=1–24% positive tumour cells, 2+=25–75% positive tumour cells and 3+=>75% positive tumour cells. In control experiments, the NESP55 antiserum was adsorbed with a synthetic peptide fragment of human NESP55 (GPIPIRRH), at 0.01–1 *μ*M overnight prior to immunocytochemical staining.

### Western blot

Fresh tumour tissues were snap frozen in liquid nitrogen and stored at −140°C until preparation. Frozen tissue (approximately 20 mg) was homogenised in 10 mM potassium phosphate buffer, pH 6.8, containing 1 mM EDTA, 10 mM 3-(3-cholamidopropyl) dimethylammonio 1-propane sulphate (CHAPS), 1 *μ*g ml^−1^ aprotinin, and 10 *μ*g ml^−1^ each of leupeptin and pepstatin and 1 mg ml^−1^ of 4-(2-aminoethyl)-benzenesulphonyl fluoride (Pefablock® SC, Boehringer Mannheim, Mannheim, Germany). Homogenates were sonicated twice for 15 s, followed by centrifugation for 10 min at 10 000 **g**. The clear supernatant was withdrawn, assayed for protein content according to Bradford and stored at −80°C. Aliquots of proteins (20 *μ*g) were diluted in NuPage™ LDS sample buffer (cat. no. NP 0007, In Vitrogen, Carlsbad, CA, USA). Reducing agent was added, followed by denaturation at 70°C for 10 min and electrophoresis on precast polyacrylamide gels (10% NuPage™ Bis-Tris-gels, cat. no. NP 0301 In Vitrogen) using NuPage™ MOPS SDS (cat. no. NP 0001, In Vitrogen) as running buffer. The proteins were transferred to polyvinyl difluoride (PVDF) membranes (cat. no. 43660, BDH 4 Q Poole, UK) using the NOVEX blotting system. Membranes were incubated with rabbit polyclonal anti-NESP55 antibody (Ischia *et al*, 1997) at 4°C overnight followed by alkaline phosphatase-conjugated goat anti-rabbit antibody (cat. no. AC31RL, Tropix, Applied Biosystems MSC 050, Bedford, MA, USA) and CDP-Star (Tropix, Applied Biosystems) as substrate. Membranes were exposed to ECL film (RPN 3103K, Amersham Pharmacia Biotech, Buckinghamshire, UK) at room temperature for 10–300 s. Molecular weight markers (SeeBlue™, LC 5625, In Vitrogen) were used to calculate the apparent size of immunoreactive proteins. In control experiments, the NESP55 antiserum was adsorbed with a synthetic peptide fragment of human NESP55 (GPIPIRRH) at 1 *μ*M overnight prior to incubation with membranes.

### Northern blot

Total RNA was extracted from tumour tissue by the acid guanidinium thiocyanate method. Total RNA was size fractionated on 1% agarose gels with 2.2M formaldehyde and transferred to nylon membranes (Boehringer Mannheim, Darmstadt, Germany). RNA was crosslinked to membranes by UV-light (Stratagene, CA, USA). Membranes were prehybridised in SSC buffer containing 50% formamide for 1–2 h at 60°C and hybridised overnight at 60°C with a NESP55 antisense probe labelled with [*α*-^32^ P]-UTP. After stringent washing, membranes were exposed to imaging plates (K screen) at room temperature and read in a Molecular Imager^R^ FX (Bio-Rad, CA, USA). The NESP55 probe was a 266-bp fragment of human NESP55 (nucleotides 1090–1356, accession number AJ009849) generated by PCR-amplification of a c-DNA fragment from a human pheochromocytoma subcloned into pPCR-Script™ Amp SK (+) cloning vector (Stratagene). The identity of the cloned NESP55 cDNA fragment was confirmed by sequence analysis. Radiolabelled antisense probe was generated using T3 RNA polymerase.

## RESULTS

### Immunocytochemical localisation of NESP55 and CgA in normal gastrointestinal tract, pancreas and adrenal gland

#### Gastrointestinal tract

NESP55 labelling was observed over a few cells in the mucosa of the gastric antrum. Labelled cells were located in the lower half of the antral glands. No NESP55 labelling was observed in the mucosa of the small intestine and colon. In the submucous and myenteric plexa, a few NESP55-labelled ganglion cells were observed. Numerous CgA-positive endocrine cells were observed in the mucosa at all levels of the gastrointestinal tract. A few CgA-positive nerve fibres and ganglion cells were observed in the myenteric and submucous nerve plexa at all levels of the gastrointestinal tract.

#### Pancreas

**Figure 1:**
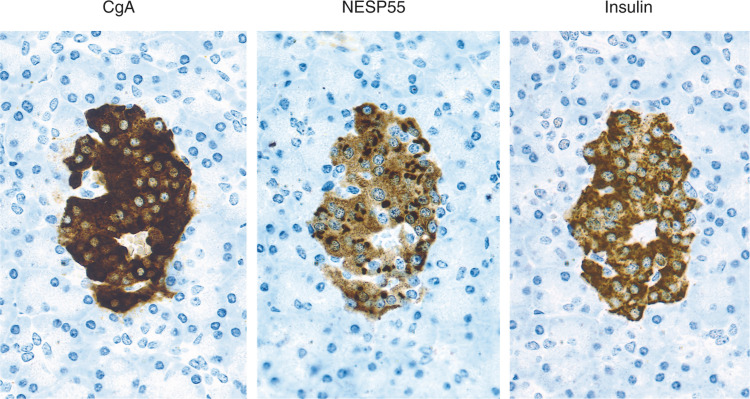
Immunocytochemical demonstration of CgA, NESP55 and insulin in a pancreatic islet. Consecutive sections were stained with an indirect immunoperoxidase technique (EnVision+™). The majority of pancreatic *β*-cells (insulin positive) are also positive for CgA and NESP55.

Neuroendocrine secretory protein 55 labelling was observed in pancreatic islets, while exocrine parenchyma was devoid of labelling. The majority of NESP55 labelling was located in beta-cells, as evident from consecutive staining for NESP55, CgA and insulin (Figure 1

). The NESP55 staining pattern was cytoplasmic, with an accumulation of labelling in the perinuclear region. All labelling was abolished after adsorption of NESP55 antiserum with 1 *μ*M synthetic human NESP55 peptide. Chromogranin A labelling was also located to pancreatic islets, with no labelling over exocrine parenchyma. The CgA labelling in pancreatic islets was cytoplasmic with a characteristic granular pattern (Figure 1).

#### Adrenal gland

**Figure 2:**
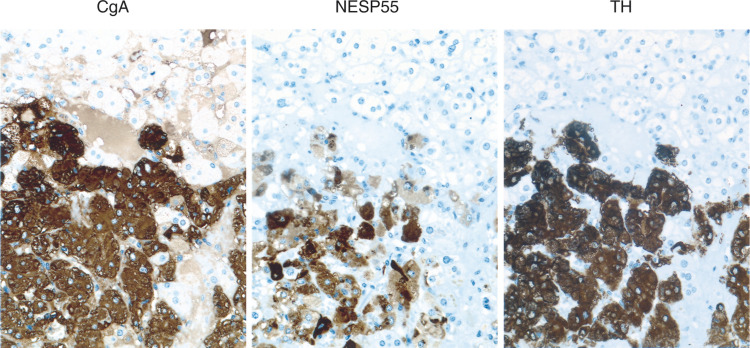
Immunocytochemical demonstration of CgA, NESP55 and TH in normal adrenal medulla. Consecutive sections were stained with an indirect immunoperoxidase technique (EnVision+™). The majority of chromaffin cells are positive for CgA, NESP55 and TH.

The chromaffin cells in the adrenal medulla were labelled by the NESP55 antibody, while none of the parenchymal cells of the adrenal cortex were labelled. The NESP55 labelling pattern in chromaffin cells was cytoplasmic, with an accumulation of labelling in the perinuclear region. All labelling was abolished after adsorption of NESP55 antiserum with 1 *μ*M synthetic human NESP55 peptide. Chromogranin A was also located to the adrenal medulla and showed a characteristic granular labelling pattern in the chromaffin cells (Figure 2

).

### Immunocytochemical localisation of NESP55 and CgA in endocrine and nonendocrine tumours of the gastrointestinal tract, pancreas and adrenal

#### Ileal endocrine tumours

A total of 15 tumours were analysed. Tumours were classified according to WHO 2000 (Socia *et al*, 2000) as being well-differentiated endocrine carcinomas (malignant carcinoids). All tumours had metastatic spread to regional lymph nodes and/or liver. All patients had a typical midgut carcinoid syndrome with overproduction of serotonin. Immunocytochemical analysis did not demonstrate any NESP55 labelling in the ileal carcinoids (0 out of 15), while all tumours (15 out of 15) were strongly positive for CgA (Table 2

**Table 2:** Immunocytochemical demonstration of NESP55 and CgA in 118 endocrine and nonendocrine tumours

**Tumour type**	**Case no.**	**NESP55**	**CgA**
Ileal endocrine tumours	(1–15)	**0/15**(15, 0, 0, 0)	**15/15**(0, 0, 0, 15)
Pancreatic endocrine tumours	(16–40)	**14/25** (11, 6, 3, 5)	**25/25** (0, 0, 2, 23)
Neuroblastomas	(41–54)	**7/14** (7, 6, 1, 0)	**14/14** (0, 0, 1, 13)
Pheochromocytomas	(55–73)	**19/19** (0, 1, 2, 16)	**19/19** (0, 0, 0, 19)
Adrenocortical adenomas	(74–88)	**0/15** (15, 0, 0, 0)	**0/15** (15, 0, 0, 0)
Gastric carcinomas	(89–94)	**1/6** (5, 1, 0, 0)	**0/6** (6, 0, 0, 0)
Colorectal carcinomas	(95–103)	**0/9** (9, 0, 0, 0)	**0/9** (9, 0, 0, 0)
Pancreatic carcinomas	(104–118)	**1/15** (14, 1, 0, 0)	**0/15** (15, 0, 0, 0)

The results are given as the number of positive tumours/total number of tumours (in bold). Tumours were divided into four categories, and the number of tumours in each category is given in parentheses (0, 1+, 2+, 3+). The categories were as follows: 0 <1% positive cells, 1+=1–24% positive cells, 2+=25–75% positive cells, 3+=>75% positive cells.

).

#### Pancreatic endocrine tumours

**Figure 3:**
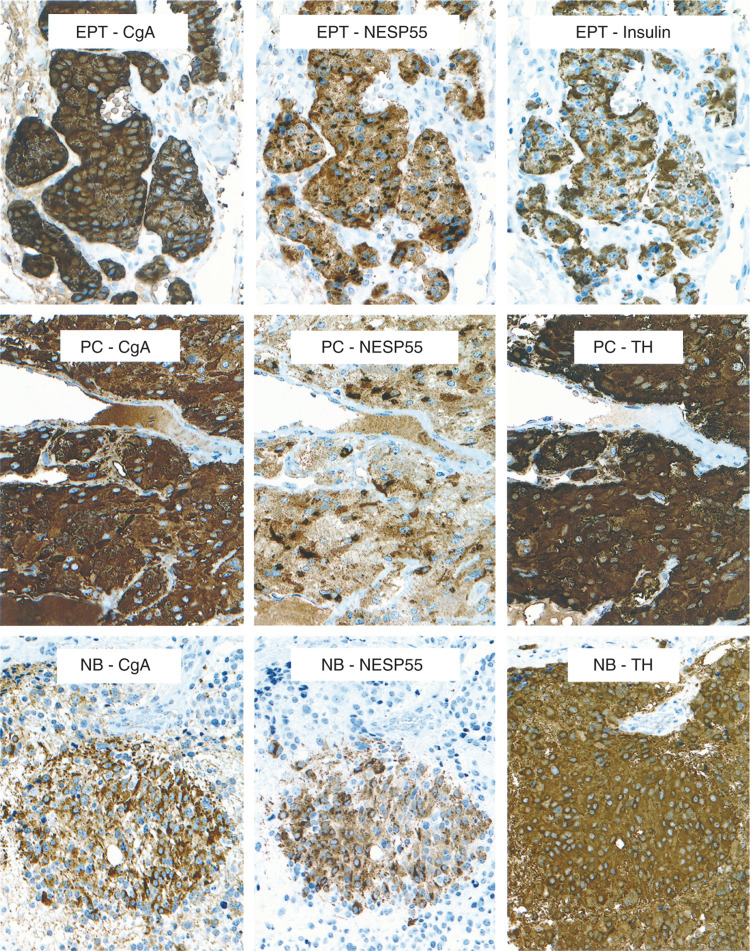
Immunocytochemical demonstration of CgA and NESP55 in a pancreatic endocrine tumour (EPT, malignant insulinoma), an adrenal pheochromocytoma (PC) and a neuroblastoma (NB). Consecutive sections were stained with an indirect immunoperoxidase technique (EnVision+™). The majority of tumour cells are positive for CgA and NESP55. TH=tyrosine hydroxylase.

A total of 25 tumours were analysed and classified according to WHO 2000. The criteria for malignancy was invasion of contiguous structures or documented metastases. The tumour material was divided into the following categories: (1) 12 well-differentiated endocrine tumours, including six insulinomas, one glucagonoma, one gastrinoma (associated with MEN 1 syndrome) and four nonfunctioning tumours; (2) 13 well-differentiated endocrine carcinomas, including two insulinomas, one gastrinoma, one glucagonoma and nine nonfunctioning tumours, (one was associated with MEN 1 syndrome). Immunocytochemical analysis demonstrated NESP55 labelling in 14 out of 25 tumours. In the group of benign tumours (well-differentiated endocrine tumours) positive NESP55 labelling was observed in eight out of 12 tumours. Extensive NESP55 labelling was observed in five out of six insulinomas and two out of four nonfunctioning tumours. Focal NESP55 labelling was observed in one out of one gastrinoma. All tumours (12 out of 12) were strongly positive for CgA. In the group of malignant tumours (well-differentiated endocrine carcinomas) positive NESP55 labelling was observed in six out of 13 carcinomas. Extensive NESP55 labelling was observed in one out of two insulinomas, while focal NESP55 labelling was observed in one out of two insulinomas and four out of nine nonfunctioning carcinomas. All carcinomas (12 out of 12) were strongly positive for CgA. Neuroendocrine secretory protein 55 labelling in benign and malignant tumours was cytoplasmic with a strong perinuclear accumulation. In a small population of tumour cells, the cytoplasmic NESP55 labelling was granular, and similar to that of chromogranin A (Figures 3

and 4

**Figure 4:**
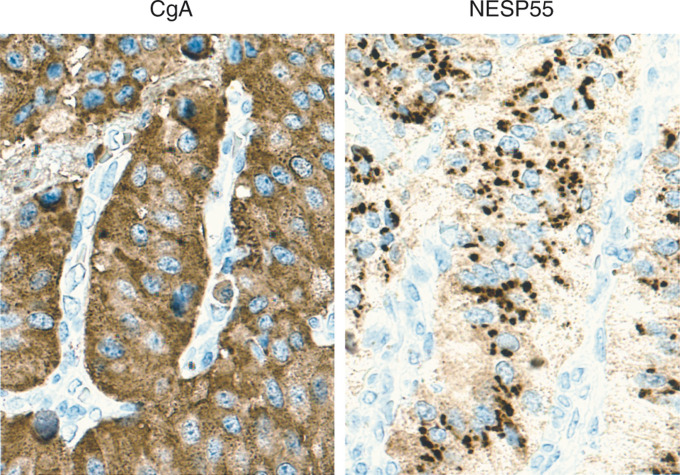
Immunocytochemical demonstration of CgA and NESP55 in a pancreatic endocrine tumour (EPT, malignant insulinoma). Chromogranin A antibodies gave a granular staining of all cytoplasm in tumour cells. NESP55 antibodies gave a cytoplasmic staining of tumour cells that was partly granular and concentrated to the perinuclear region. Indirect immunoperoxidase staining (EnVision+™).

). All labelling was abolished after adsorption of NESP55 antiserum with 1 *μ*M synthetic human NESP55 peptide. A summary of clinical and immunocytochemical results on endocrine pancreatic tumours is given in Tables 2 and 3

**Table 3:** Clinical and immunocytochemical findings in 25 pancreatic endocrine tumours

					**Metastases**			**Clinical outcome**				**ICC findings**	
	**Case no.**	**Age**	**Sex**	**Hormonal Syndrome**	**Lgl**	**Liver**	**Years**	**NED**	**AWD**	**DWD**	**DOD**	**NESP55**	**CgA**
*Well-differentiated endocrine tumour*													
Insulinoma	**16**	62	M	Yes	—	—	1	X	0	0	0	2+	3+
	**17**	80	F	Yes	—	—	8	X	0	0	0	3+	3+
	**18**	17	F	Yes	—	—	3	X	0	0	0	3+	3+
	**19**	32	F	Yes	—	—	1	X	0	0	0	—	3+
	**20**	83	F	Yes	—	—	1	X	0	0	0	3+	3+
	**21^a^**	68	F	Yes	—	—	7	X	0	0	0	3+	3+
Glucagonoma	**22**	50	F	Yes	—	—	7	X	0	0	0	—	3+
Gastrinoma	**23^a^**	55	M	Yes	—	—	3	X	0	0	0	1+	3+
Nonfunctioning	**24**	63	F	No	—	—	4	X	0	0	0	—	3+
	**25**	63	M	No	—	—	1	X	0	0	0	2+	3+
	**26**	25	M	No	—	—	11	X	0	0	0	—	3+
	**27**	76	F	No	—	—	4	X	0	0	0	2+	3+
*Well-differentiated endocrine carcinoma*													
Insulinoma	**28**	62	M	Yes	+	—	<1	0	0	X^b^	0	3+	3+
	**29**	69	M	Yes	—	+	<1	0	0	X^c^	0	1+	3+
Glucagonoma	**30**	49	M	Yes	+	—	6	X	0	0	0	—	3+
Gastrinoma	**31**	30	F	Yes	—	+	7	0	0	0	X	—	3+
Nonfunctioning	**32**	48	M	No	—d	—	2	0	X	0	0	1+	3+
	**33^a^**	55	M	No	+	—	2	0	X	0	0	—	2+
	**34**	53	M	No	+	+	3	X	0	0	0	1+	3+
	**35**	40	M	No	—	+	4	X^e^	0	0	0	1+	3+
	**36**	49	M	No	+	—	4	0	X	0	0	1+	3+
	**37**	47	M	No	+	—	1	0	0	0	X	—	2+
	**38^f^**	55	F	No	+	—	5	X	0	0	0	—	3+
	**39**	58	F	No	+	+	5	0	X	0	0	—	3+
	**40**	45	F	No	+	+	7	X^g^	0	0	0	—	3+

Age=age at diagnosis and primary surgery. F=female, M=male. Metastases: Lgl=lymph node metastases, liver=liver metastases. Clinical outcome: Years=observation time. X indicates outcome at follow up, which was categorized as NED=alive, no evidence of disease. AWD=alive with disease. DWD=dead with disease. DOD=dead of disease. ICC findings: The categories were as follows: 0=<1% positive cells, 1+=1–24% positive cells, 2+=25–75% positive cells, 3+=>75% positive cells.

a MEN 1 syndrome.

b Died postoperatively because of surgical complications.

c Died from metastatic rectal carcinoma.

d Tumour invading large vessels.

e Treated with orthotopic liver transplantation (OLT) following diagnosis, treated for local recurrence 3 years post-OLT, and treated for adrenal metastases 4 years post-OLT.

f Von Hippel–Lindau syndrome.

g Treated with orthotopic liver transplantation (OLT), 5 years after primary surgery because of liver metastases.

.

#### Neuroblastomas

A total of 14 malignant adrenal neuroblastomas were examined. Clinical staging of tumours was according to the International Neuroblastoma Staging System (INSS) (Brodeur *et al*, 1993). Neuroendocrine secretory protein 55 labelling was detected in seven out of 14 neuroblastomas. The labelling of tumours was sparse to moderate (<75% of tumour cells; 1+, 2+). Labelling was granular and confined to the cytoplasm of tumour cells. All labelling was abolished after adsorption of NESP55 antiserum with 1 *μ*M synthetic human NESP55 peptide. Chromogranin A labelling was detected in 14 out of 14 neuroblastomas. Labelling of tumour cells was strong (>75% of tumour cells; 3+) in the majority of tumours (13 out of 14). The labelling pattern of NESP55, CgA and TH was compared in consecutive sections from some tumours. Tyrosine hydroxylase labelling was extensive and present in all tumour cells, while CgA and NESP55 labelling was more limited and present in a subpopulation of tumour cells (Figure 3). A summary of clinical and immunocytochemical results on neuroblastomas is given in Tables 2 and 4

**Table 4:** Clinical and immunocytochemical findings in 14 neuroblastomas

										Clinical outcome				ICC findings	
**Case no.**	**Age**	**Sex**	**Stage**	**N-*myc***	**NSE**	**HVA**	**VMA**	**DA**	**Years**	**NED**	**AWD**	**DWD**	**DOD**	**NESP55**	**CgA**
**41**	<1	M	1	ND	ND	56	35	ND	12	X	0	0	0	—	3+
**42**	<1	M	2B	—	14	95	112	430	5	X	0	0	0	—	3+
**43**	<1	F	2B	>10	13	30	19	1358	5	X	0	0	0	+1	3+
**44**	3	M	3	>10	210	180	51	40 079	2	0	0	0	X	+1	3+
**45**	3	F	3	ND	ND	150	47	41 608	1	0	0	0	X	+1	3+
**46**	2	F	3	ND	ND	36	6.9	ND	9	X	0	0	0	+1	3+
**47**	1	F	3	ND	ND	440	343	9277	8	X	0	0	0	+1	3+
**48**	1	F	3	ND	68	130	300	ND	14	X	0	0	0	+2	3+
**49**	4	M	4	>70	ND	3.7	13	ND	1	0	0	0	X	—	2+
**50**	2	F	4	—	200	87	6.2	ND	1	0	0	0	X	—	3+
**51**	1	M	4	ND	ND	85	14	ND	2	0	0	0	X	—	3+
**52**	<1	F	4	>10	12	17	15	1665	12	X	0	0	0	—	3+
**53**	5	M	4	—	270	30	17	2454	1	0	0	0	X	+1	3+
**54**	3	M	4	ND	ND	318	498	ND	3	0	0	0	X	—	3+

Age=age at diagnosis . F=female, M=male. Stage according to the International Neuroblastoma Staging System (INSS). N-myc=amplification of N-*myc* gene. NSE=neuron-specific enolase. HVA=homovanillic acid. VMA=vanillylmandelic acid. DA=dopamine. Years=observation time. X indicates outcome at follow up, which was categorized as NED=alive, no evidence of disease. AWD=alive with disease. DWD=Dead with disease. DOD=dead of disease. ICC findings: The categories were as follows: 0=<1% positive cells, 1+=1–24% positive cells, 2+=25–75% positive cells, 3+=>75% positive cells. ND=not determined.

.

#### Pheochromocytomas

A total of 19 tumours were examined and classified according to WHO 2000: pheochromocytomas (adrenal paragangliomas), of which 14 were benign and five malignant with metastatic spread (Table 5

**Table 5:** Clinical and immunocytochemical findings in 19 pheochromocytomas

						**Clinical outcome**				**ICC findings**	
**Case no.**	**Age**	**Sex**	**Malignant/benign**	**Hormonal syndrome**	**Years**	**NED**	**AWD**	**DWD**	**DOD**	**NESP55**	**CgA**
**55**	47	F	Benign	Yes	8	X	0	0	0	3+	3+
**56**	53	M	Benign	Yes	7	X	0	0	0	3+	3+
**57**	27	F	Benign	Yes	3	X	0	0	0	3+	3+
**58**	48	M	Benign	Yes	8	X	0	0	0	3+	3+
**59**	52	M	Benign	Yes	4	X	0	0	0	3+	3+
**60**	73	M	Benign	Yes	1	X	0	0	0	3+	3+
**61**	52	M	Benign	Yes	1	X	0	0	0	3+	3+
**62^a^**	38	F	Benign	No	2	X	0	0	0	3+	3+
**63**	47	F	Benign	Yes	3	X	0	0	0	2+	3+
**64**	20	F	Benign	Yes	2	X	0	0	0	3+	3+
**65^b^**	18	M	Benign	Yes	1	X	0	0	0	3+	3+
**66^c^**	20	F	Benign	Yes	7	X	0	0	0	2+	3+
**67**	61	F	Benign	Yes	4	X	0	0	0	1+	3+
**68**	56	M	Benign	Yes	2	X	0	0	0	3+	3+
**69**	76	F	Malignant	Yes	8	0	0	X	0	3+	3+
**70**	36	F	Malignant	No	23	0	0	0	X	3+	3+
**71**	55	M	Malignant	No	14	X	0	0	0	3+	3+
**72**	58	M	Malignant	Yes	1	0	0	X	0	3+	3+
**73**	61	M	Malignant	Yes	9	0	0	X	0	3+	3+

Age=age at diagnosis and primary surgery. F=female, M=male. Clinical outcome: Years=observation time. X indicates outcome at follow up, which was categorized as NED=alive, no evidence of disease. AWD=alive with disease. DWD=dead with disease. DOD=dead of disease. ICC findings: The categories were as follows: 0=<1% positive cells, 1+=1–24% positive cells, 2+=25–75% positive cells, 3+=>75% positive cells.

a Turner's syndrome,

b Familial pheochromocytoma, genetic defect unknown.

c MEN 2B syndrome.

). Hormonal symptoms were present in 16 out of 19 patients. Immunocytochemical results demonstrated NESP55-labelling in all 19 pheochromocytomas, both benign and malignant tumours. The staining was localised to the cytoplasm of tumour cells, with a perinuclear accumulation (Figure 3). All labelling was abolished after adsorption of NESP55 antiserum with 1 *μ*M synthetic human NESP55 peptide. All tumours (19 out of 19) were strongly positive for CgA, with a granular cytoplasmic staining. A summary of clinical and immunocytochemical results on pheochromocytomas is given in Tables 2 and 5.

#### Adrenocortical adenomas

A total of 15 adrenocortical tumours were examined, and classified as benign adrenocortical adenomas according to Weiss (1984): four nonfunctioning tumours, four Conn tumours and seven tumours with Cushing syndrome. Immunocytochemical analysis revealed no NESP55 or CgA labelling in any of the tumours (Table 2).

#### Nonendocrine tumours of the gastrointestinal tract

The following nonendocrine tumours of the gastrointestinal tract were examined: six gastric adenocarcinomas, nine colorectal adenocarcinomas and 15 pancreatic carcinomas. Immunocytochemical results demonstrated no NESP55 labelling in the majority of the tumours (28 out of 30). One gastric adenocarcinoma, and one pancreatic adenocarcinoma contained scattered NESP55-labelled cells. CgA was absent in all of the tumours (30 out of 30) (Table 2).

### Characterisation of NESP55 protein in endocrine tumours by Western blot

**Figure 5:**
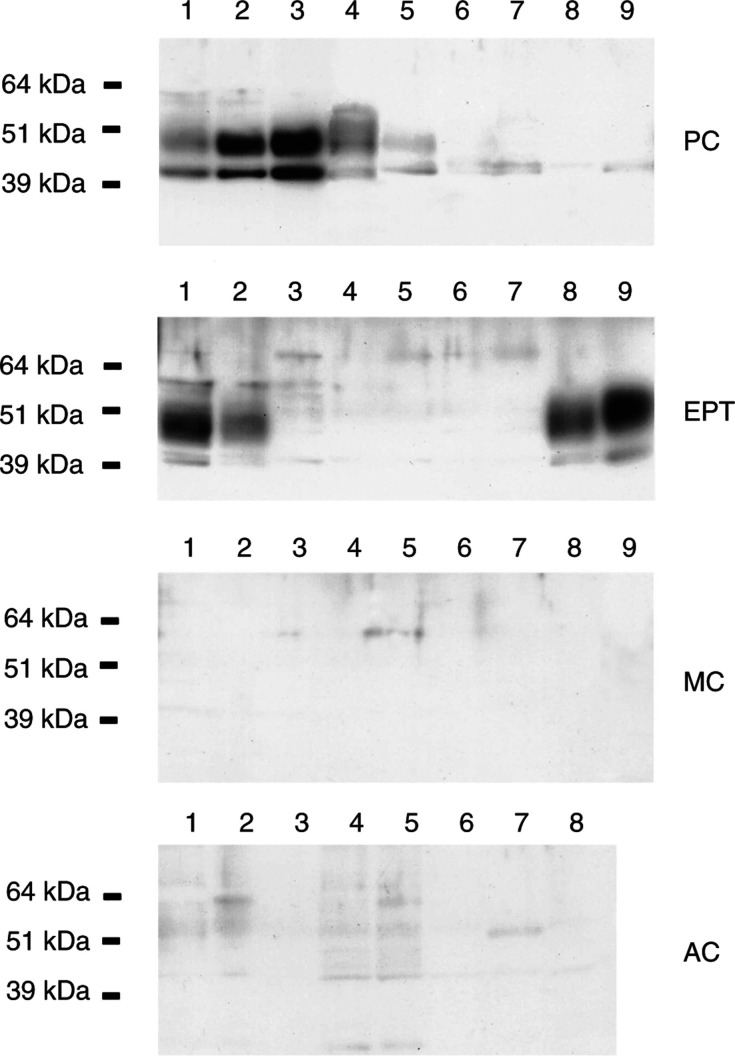
Expression of NESP55 in adrenal pheochromocytomas (PC, *n*=9, all benign tumours), pancreatic endocrine tumours (EPT, *n*=9, benign insulinomas lanes 1, 8, 9; malignant insulinoma lane 2, malignant nonfunctioning tumours lanes 3–7), ileal (midgut) carcinoids (MC, *n*=9) and gastrointestinal adenocarcinomas (AC, *n*=8) analysed by Western blot. The NESP55 immunoreactive protein migrated as a major band of 45–55 kDa and a minor band at 40 kDa.

Western blot analysis demonstrated NESP55 expression in five out of nine adrenal pheochromocytomas (all benign tumours), and in four out of nine pancreatic endocrine tumours. All NESP55-expressing endocrine pancreatic tumours were insulin-producing (1 malignant and three benign insulinomas), while all NESP55-negative tumours were nonfunctioning malignant tumours. The NESP55 immunoreactive protein in pheochromocytomas and endocrine pancreatic tumours migrated as a major band of 45–55 kDa. In some tumours, an additional minor band of approximately 40 kDa was detected. Adsorption of NESP55 antiserum with 1 *μ*M of synthetic human NESP55 peptide (GPIPIRRH) totally abolished both NESP55 bands. None of the ileal carcinoids (*n*=9) and none of the gastrointestinal adenocarcinomas (*n*=8) expressed NESP55 protein (Figures 5

and 6

**Figure 6:**
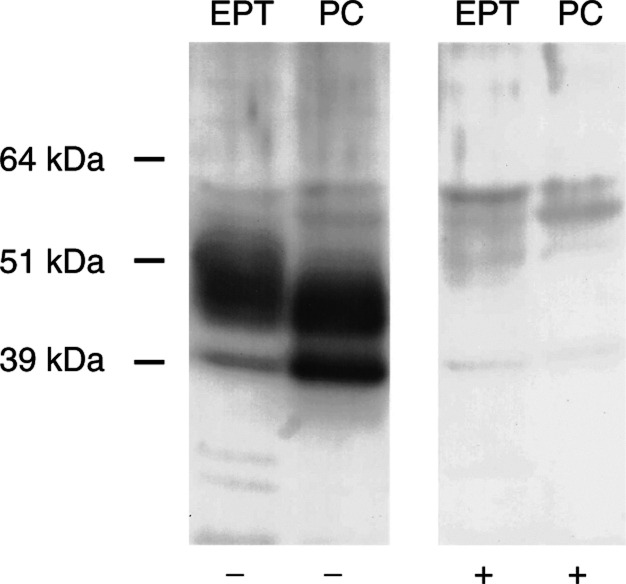
Effect of synthetic NESP55 peptide on Western blot analysis of NESP55 in EPT (benign insulinoma) and adrenal pheochromocytoma (PC, benign tumour). Neuroendocrine secretory protein 55 antiserum was adsorbed with a synthetic fragment of human NESP55 (GPIPIRRH) at 1 *μ*M overnight prior to incubation. In unadsorbed blots (−), the NESP55 immunoreactive protein migrated as a major band of 45–55 kDa and a minor band at 40 kDa. In adsorbed blots (+), all NESP55 immunolabelling was abolished.

).

### Analysis of NESP55 transcripts in endocrine tumours by Northern blot

**Figure 7:**
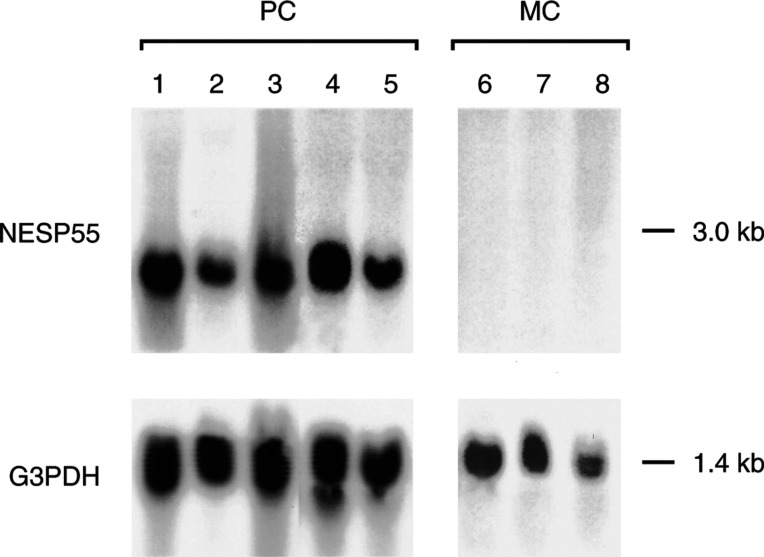
Expression of NESP55 in adrenal pheochromocytomas (PC, benign tumours, lanes 1–5) and ileal (midgut) carcinoids (MC, lanes 6–8) analysed by Northern blot. A transcript of approximately 3.0 kb was detected.

Northern blot analysis of NESP55 mRNA demonstrated a transcript of approximately 3 kb in adrenal pheochromocytomas (*n*=5). No NESP55 transcripts were detected in ileal endocrine tumours (*n*=3) (Figure 7

).

## DISCUSSION

Neuroendocrine tumours are characterised by their content of secretory granules and vesicles. Components from these organelles are frequently used to identify and classify neuroendocrine tumours. Vesicle membrane proteins, such as synaptophysin and SV2, are used as general markers since they are present in virtually all neuroendocrine tumours (Jakobsen *et al*, 2002). Other vesicle membrane proteins, for example, VMAT 1 and 2, are expressed in a limited number of neuroendocrine tumours, but can be used to identify subgroups of tumours, and may indicate potential treatment strategies (Jakobsen *et al*, 2001). Granule proteins located to dense core, for example, chromogranins, are also used as general markers of neuroendocrine differentiation since almost all neuroendocrine tumours express CgA. In the present study, we have evaluated the expression of NESP55 in human endocrine and nonendocrine tumours and compared it with CgA expression. We found by immunocytochemistry that pancreatic endocrine tumours, pheochromocytomas and neuroblastomas expressed the NESP55 protein regardless of malignant potential and tumour stage. Western and Northern blot analysis confirmed expression of NESP55 in pancreatic endocrine tumours and pheochromocytomas. Northern blot analysis of NESP55 in tumour tissues demonstrated a single mRNA transcript of 3 kb, a pattern that was different from that observed in normal rat tissues, where five distinct transcripts have been identified (Weiss *et al*, 2000).

NESP55 could not be detected in ileal carcinoids. Thus, the expression of NESP55 was different from that of CgA, which was expressed in all neuroendocrine tumours investigated, regardless of hormone production, cellular composition or malignant potential. These results demonstrate that NESP55 is expressed by a subset of neuroendocrine tumours, and that NESP55 cannot be regarded as a general neuroendocrine tumour marker. In normal tissues, NESP55 was present in chromaffin cells of the adrenal medulla and in pancreatic islet cells. Tumours originating from these organs also expressed NESP55, reflecting their origin and cellular composition.

The function of NESP55 in neuroendocrine tumours is unknown. The role of the chromogranins in normal cells is not yet fully understood. However, it has recently been proposed that CgA regulates secretory granule biogenesis in endocrine and neuroendocrine cells (Kim *et al*, 2001). All known chromogranins including NESP55 are proteolytically processed, to give rise to smaller peptide fragments, some of which possess biological activity. We suggest that NESP55 in neuroendocrine tumours may serve a function in secretory granule formation and that proteolytically processed fragments of NESP55 are secreted and may influence tumour cell growth/secretion.

In conclusion, we have demonstrated that NESP55 is expressed in neuroendocrine tumours of the pancreas and adrenals. The distribution of NESP55 differs from that of CgA in neuroendocrine tumours. The expression of NESP55 reflects the origin and cellular composition of neuroendocrine tumours and may therefore become a valuable marker in diagnostic histopathology.
